# Synergistic effect of *Trichoderma harzianum* and chitosan nanoparticles on garlic plants in arid regions

**DOI:** 10.1186/s12870-025-07844-5

**Published:** 2025-12-12

**Authors:** Osama Abdelsalam Shalaby

**Affiliations:** Plant Production Department, Desert Research Center, Cairo, Egypt

**Keywords:** Biofertilizers, Nanomaterials, Plant biostimulants, Plant growth, Sustainable agriculture, Vegetables.

## Abstract

The intensive use of agrochemicals is not conducive to sustainable agricultural production, as it leads to soil degradation, harms soil organisms, and causes environmental pollution. Hence, adopting natural and biological practices in plant production management is a sustainable and environmentally friendly strategy. Therefore, this study investigated the effectiveness of combining *Trichoderma harzianum* inoculation and chitosan nanoparticle spraying in garlic cultivation, where two levels of *T. harzianum* inoculation (inoculated and uninoculated) and three levels of nano-chitosan spraying (0, 50, and 100 ppm) were used in an open-field experiment. Overall, *Trichoderma* fungi and chitosan nanoparticles, either alone or in combination, increased nutrient uptake, improved physiological status, promoted plant growth, and increased garlic yield. However, garlic plants inoculated with *T. harzianum* and sprayed with 100 ppm chitosan nanoparticles presented superior results over all the other treatments, increasing nutrients (N, P, K, and S), photosynthetic pigments (chlorophyll a, b, and carotenoids), and antioxidants (phenolic compounds, flavonoids, and vitamin C), resulting in significant increases in plant height (16%), bulb fresh weight (22%), bulb diameter (15%), and bulb dry weight (25%). These findings suggest that microbial and natural biostimulants, such as *Trichoderma* and chitosan, can be considered successful and sustainable agricultural practices to increase physiological performance, bioactive compound accumulation, and yield of vegetable plants.

**Supplementary Information:**

The online version contains supplementary material available at 10.1186/s12870-025-07844-5.

## Introduction

Sustainable food production faces significant challenges in achieving food security in semiarid and arid regions. Factors such as limited environmental resources, climate change, abiotic stresses, and unsound agricultural practices, particularly the overuse of agrochemicals such as pesticides and mineral fertilizers, significantly impact food quality, soil health, and environmental sustainability. As a result, there is an urgent need to implement environmentally friendly agricultural practices that reduce pollution, adapt to climate change, pave the way for sustainable production, and improve food quality [1–3]. Although the overuse of agrochemicals is harmful, farmers use large quantities of chemical fertilizers and pesticides to ensure abundant production without affecting human health, soil organisms, the environment, or sustainability. Therefore, to reduce their detrimental effects on humans and the environment, biofertilizers and natural-origin materials should be used [4, 5]. Chitosan and *Trichoderma* fungi are examples of natural and microbial biostimulants that can enhance physiological responses, metabolic processes, root structure and function, nutrient utilization, photosynthetic efficiency, and antioxidant defense systems, all of which can increase plant growth and productivity [2, 6]. Ecological agriculture aims to restrict the use of synthetic chemicals, which may reduce plant productivity, necessitating additional environmental practices to compensate for this deficiency. Biofertilizers are an eco-friendly and sustainable alternative method that reduces the use of agrochemicals, improves the nutritional status and physicochemical properties of soil, controls disease, promotes growth, and increases crop yield [1, 7].

Beneficial microbe applications are sustainable agricultural practices that reduce the use of synthetic chemicals. *Trichoderma* fungi provide a successful model for interactions between plants and beneficial microorganisms, effectively contributing to pathogen control, nutrient availability, growth stimulation, and stress resistance [8]. The genus *Trichoderma* includes more than 100 species that are capable of growing under various conditions and affecting all stages of plant growth. These fungi secrete growth-promoting substances, regulate phytohormone synthesis, improve root structure and function, increase photosynthetic efficiency, induce stress resistance, and combat plant diseases, all of which contribute to increased plant growth and productivity [5, 9]. By establishing a symbiotic relationship with roots, plants inoculated with *Trichoderma* fungi via seed priming, soil application, or foliar spraying act as plant biostimulants and pesticides [4, 5, 10, 11]. *Trichoderma* fungi improve soil fertility and nutrient availability by decomposing organic materials and solubilizing soil nutrients, which reduces mineral fertilizer use [1, 4, 12, 13]. In the same setting, phosphate-solubilizing *Trichoderma harzianum* enhances the availability of soil phosphorus, increasing plant phosphorus content by 34% [11]. *T. harzianum* improves soil fertility by activating soil enzymes (such as urease, phosphatase, phytase, sucrose, cellulase, and catalase) and secreting organic acids (such as citric, acetic, lactic, formic, and oxalic), which improve soil nutrient cycling and metabolism and facilitate nutrient uptake. Therefore, *Trichoderma* fungi are used as biofertilizers and biopesticides [14, 15].

*Trichoderma* spp inoculation improves physiological responses, growth performance, and crop yield by producing growth-promoting substances and defense-related enzymes [12]; reduces the severity of plant diseases by reducing pathogen numbers and stimulating systemic resistance [4, 12, 16]; improves root structure and function by secreting plant growth regulators and organic acids [1]; and increases plant stress tolerance [17]. *Trichoderma* inoculation also alters the physical and chemical properties of the soil, increases microbial activity, suppresses soil-borne diseases, and activates defense mechanisms, thereby reducing disease incidence and fungicide use while increasing yields of garlic and potatoes [18, 19]. *Trichoderma*-inoculated plants showed improved growth parameters, nutrient content, chlorophyll levels, and increased tomato and onion yield [1, 17]; increased photosynthetic efficiency, antioxidant activity (vitamin C, phenolic, and proline), and growth and yield parameters (plant length, fresh and dry weight, fruit weight, and diameter) in tomato plants [9, 12]; and enhanced nutrient availability (N, P, and K), nutrient acquisition, and growth potential, soluble sugar accumulation, and antioxidant enzyme activity in grass plants [20]. By improving soil structure and the abundance and activity of beneficial microbes, *T. harzianum* increased nutrient availability and uptake, as well as the growth, yield, and quality of *Bupleurum chinense* plants [7]. It also enhanced photosynthetic pigments, antioxidants (proline, ascorbic acid, catalase, and flavonoids), nutrient content, and yield (bulb diameter and weight) in onion plants [2] and increased phenols, flavonoids, and indole acetic acid in onions [17].

Therefore, *Trichoderma harzianum* inoculation could be an effective strategy to reduce soil-borne pathogens and increase the abundance of beneficial microbes, with plants showing a 51% relative control efficacy and a 58% increase in wheat yield [21]. *Trichoderma* application also significantly reduces the adverse effects of abiotic stress in plants by enhancing enzymatic and nonenzymatic antioxidants, lowering toxic compounds such as H₂O₂ and MDA, increasing photosynthetic pigments, including chlorophyll a, chlorophyll b, and carotenoids, and improving nutrient absorption. This results in improved mung bean parameters (fresh and dry weight, root length, root biomass, and seed yield) under heavy metal stress [15], increased Indian mustard yield under salt stress [22], enhanced *Cucurbita pepo* growth performance under both normal and saline conditions [10], and better lettuce growth resilience under heat stress [13].

Nanotechnology is an emerging field for manufacturing nanomaterials measuring less than 100 nm. Owing to their size, cationic nature, optical properties, and high surface-to-volume ratio, nanoparticles have unique functional features, which qualify them for various key applications in diverse scientific fields. This technology enables efficient, practical, and sustainable agricultural practices by providing nanomaterials with superior functional properties in terms of shape, size, and operating efficiency. Compared with traditional materials, nanomaterials have many agricultural applications, including fertilizers and pesticides, due to their high effectiveness (easy absorption and transport within plants) and low environmental impact [23–25]. Recent studies have indicated that modern agricultural practices using nanoparticles limit the need for fertilizers and pesticides, thereby reducing pollution, conserving natural resources, ensuring sustainable food production, enhancing plant growth, controlling plant diseases, and increasing productivity [26, 27].

Chitosan is a polymer found naturally in seashells and is produced by the deacetylation of chitin. It has specific chemical and biological features, such as biodegradability, ability to act as an antioxidant, ability to stimulate growth, and antimicrobial ability. Therefore, it is an effective plant stimulant and biological control agent, as it enhances plant growth, activates defense responses, and controls plant diseases [6, 23, 24, 28]. The application of chitosan and nanochitosan via seed priming, soil application, or foliar spraying markedly enhances plant growth by augmenting soil microbial communities and activity, thereby improving soil fertility and increasing nutrient availability and uptake [27]. However, owing to their greater surface area, permeability, metabolic activity, antipathogenic potential, and smaller size, chitosan nanoparticles have outperformed conventional chitosan [25–27, 29]. Chitosan nanoparticles act as natural biostimulants that increase plant productivity under normal and stress conditions by inducing the antioxidant defense system and reducing the production of toxic compounds [30], and can be used in organic farming as an economically sustainable approach [25, 26]. Compared with those under conventional chitosan treatment, spraying plants with chitosan nanoparticles induced innate immune responses and increased the mineral content, growth, and yield parameters of finger millet [26]. Additionally, they promote bean growth and yield parameters, including plant height, pod number, and seed yield, under salt stress by increasing the contents of nutrients (N, P, and K), photosynthetic pigments (chlorophylls and carotenoids), and antioxidants (phenols, proline, ascorbic acid, and antioxidant enzymes) while reducing H₂O₂ and malondialdehyde [6]. Nanochitosan improved the physiological status of *Salvia abrotanoides* under drought stress by increasing soluble sugars, proline, chlorophyll, carotenoids, phenols, relative water content, and antioxidant enzyme activity [31]. It also alleviated heavy metal stress in tomato plants by increasing nutrient uptake, chlorophyll content, photosynthetic efficiency, and antioxidant activity while reducing toxic compound accumulation [25].

Spraying with chitosan nanoparticles enhances tomato productivity (fruit weight and diameter), bioactive compounds (phenols, flavonoids, and vitamin C), and antioxidant activity [30]; it also increases leaf area, chlorophyll, photosynthesis, plant biomass, wheat yield [24], total chlorophyll and phytohormones (indoleacetic acid and gibberellic acid) and rice yield [27]. Presowing lupine seeds with chitosan nanoparticles increased plant growth (shoot length and plant weight), yield (pod number and seed weight), chlorophyll, proline, phenol, flavonoid, and indoleacetic acid contents [29], and improved physiological responses in mung bean plants under salt stress by activating tolerance mechanisms such as the antioxidant defense system (proline, ascorbic acid, flavonoids, and enzymes), increasing the chlorophyll content, and reducing H_2_O_2_ and MDA accumulation [32]. However, the effectiveness of chitosan nanoparticles is dose dependent; low concentrations have positive effects, and high concentrations can be toxic, cause plant damage, and reduce yield [31].

Chitosan nanoparticles exhibited a compatible interaction with *Trichoderma* fungi, significantly improving their activity. In other words, chitosan nanoparticles exerted a synergistic stimulatory effect on *Trichoderma* spp., effectively suppressing soil-borne pathogenic fungi and improving plant health [33, 34]. Garlic (*Allium sativum* L.) is one of the oldest vegetables in the Alliaceae family. It is commonly used as a food for its spicy flavor and nutritional benefits and for medicinal purposes because of its bioactive substances, including antioxidants, amino acids, organic sulfur, polyphenols, and flavonoids, which provide numerous health benefits, such as antimicrobial and antioxidant activities. Garlic is a cash crop in Egypt cultivated for consumption and export [2, 35]. However, plants in arid and semi-arid regions face various environmental stresses that hinder growth and reduce productivity. As a result, farmers often resort to methods or substances that negatively impact crop quality and environmental resources. Thus, it is crucial to enhance garlic cultivation using natural means [36, 37]. Therefore, this study explores alternative, natural, eco-friendly methods that stimulate plant growth, increase crop yields, reduce agrochemical use, and achieve sustainability goals. Microbial inoculation provides the greatest benefits to the host plant. Although the application of *Trichoderma* spp. in plants has gained increasing interest recently, its effect on garlic growth and yield remains insufficiently studied. Nanotechnology could provide a novel platform for developing advanced materials and innovative cultivation techniques. Therefore, the novelty of this work lies in evaluating the synergistic effect of *T. harzianum* and chitosan nanoparticles on growth performance, nutrient content, physiology, and yield of garlic plants under arid conditions, which has not been comprehensively studied before.

## Materials and methods

This study investigated the effects of *Trichoderma harzianum* inoculation and chitosan nanoparticle application on the growth and yield of garlic plants in an open field of sandy soil (EC, 1.46 dS m⁻¹ and pH, 7.89) at Baloza Research Station, latitudes of 30° 07’ North and the longitude 31° 20’ East, North Sinai, Desert Research Center, during the winter seasons of 2021 and 2022. The average monthly climate data for the study area is shown in Table 1. The experiment used a randomized complete block design with six treatments, arranged in a split-plot design with three replicates. *T.* harzianum inoculation treatments (inoculated and uninoculated) were included in the main plot, whereas chitosan nanoparticle treatments (0, 50, and 100 ppm) [24, 25] were included in the subplots. Before planting, healthy, uniform garlic cloves of the Sids 40 variety were selected. During the first week of October, garlic cloves were planted 2–3 cm deep in the soil on either side of the irrigation line, 15 cm between each clove, and 75 cm between the irrigation lines. The experimental plot area was 18 m². *Trichoderma harzianum* EMCC 540 strain was obtained from the Egyptian Microbial Culture Collection (EMCC) at Cairo Microbiological Resources Centre (Cairo MIRCEN), Faculty of Agriculture, Ain Shams University (registered with the World Data Center for Microorganisms (WDCM) under number 583), https://www.10.12210/ccinfo.EMCC. For inoculation, 5 mL of inoculum (spore suspension, 10^6^ spores/mL) was placed below the cloves at planting. To confirm colonization, the treatment was repeated one month after planting using the irrigation system with a rate of 4 L ha⁻¹ of inoculant. Chitosan nanoparticles (C-NPs) were prepared via the ionic gelation method [38]. The characterization of chitosan nanoparticles was described by Ramadan et al. [38]. Forty-five days after planting, foliar spraying with chitosan nanoparticles was performed at the specified concentrations, and this process was repeated twice at one-month intervals. Tween 20 was added to spray solutions as a surfactant to improve surface coverage and retention. The same agricultural practices were applied to all the experimental plots.

**Table 1:** Average monthly climate data for the study area

Month	Temperature			Precipitation (mm) month-1	Relative Humidity (%)
	Max.	Min.	Mean		
January	16.8	9.3	13.1	26	50.8
February	18.6	10.5	14.6	13	46.9
March	18.0	10.6	14.3	13	43.7
April	24.9	15.8	20.4	0	45.5
May	26.7	18.6	22.7	0	45.5
Jun	30.2	23.2	26.7	1	47.3
July	31.9	24.5	28.2	0	49.0
August	31.8	25.8	28.8	0	51.3
Septamber	30.7	24.8	27.8	0	50.8
October	27.6	21.9	24.8	18	52.1
November	24.2	17.5	20.9	10	49.8
December	21.6	14.5	18.1	37	55.1

According to Salem et al. [39]

Yield traits

Plant samples were collected from each plot at harvest to evaluate their morphological, yield, and chemical characteristics. After the plant length (from bulb top to leaf top) and bulb diameter (using a caliper) were measured, the bulbs were separated from the leaves, and their fresh weights (FW) were determined. Then, the bulbs were divided into cloves and counted. The total soluble solids (TSS) in harvested cloves were measured via a refractometer (°Brix).

### Mineral content

The garlic samples were dried in an oven at 70 °C to a consistent weight, after which the dry weight (DW) was recorded. The samples were ground into powder. The nitrogen concentration was determined via micro-Kjeldahl digestion [40], the phosphorus concentration was determined according to Jackson [40], the potassium concentration was determined via a flame photometer [41], and the sulfur concentration was determined according to Rowell [42].

### Chlorophyll and carotenoid contents

Chlorophyll (a, b) and carotenoid contents were determined according to the methods of Arnon [43]. A total of 0.5 g of garlic leaves was homogenized in 80% acetone, and the absorbance of the extract was measured spectrophotometrically at 663, 645, and 470 nm and expressed as mg g^− 1^ FW.

### Ascorbic acid

Ascorbic acid was measured via the 2,6-dichloroindophenol titrimetric method. Garlic cloves were extracted with metaphosphoric acid, centrifuged for 10 min at 3000 × g, and then titrated with 2,6-dichlorophenol indophenol until pink [44].

### Soluble sugar

The method of Dubois et al. [45] was used to determine the soluble sugar content. 0.1 g of garlic clove was homogenized with 5 mL of 80% ethanol in a hot bath and centrifuged at 3000 rpm for 10 min. Then, 2 mL of the extract was added to 1 mL of 5% phenol and 5 mL of concentrated sulfuric acid. The absorbance of the mixture was measured at 490 nm.

### Phenols

To determine the phenolic content, 0.5 g of the garlic sample was homogenized with 5 mL of 80% methanol, centrifuged, and filtered. The supernatant was mixed with 2.5 ml of 10% Folin-Ciocalteu reagent and 2 ml of 7.5% sodium carbonate and left for 90 min. Then, the absorbance was measured at 750 nm, and the results are expressed as mg gallic acid/g DW [46].

### Flavonoids

To determine the flavonoid content according to the method of Lin and Tang [47], 0.1 mL of methanolic extract was added to a mixture of 300 µL of ethanol (95%), 40 µL of aluminum chloride (10%), 40 µL of potassium acetate (1.0 M), and 520 µL of distilled water and left at room temperature for 40 min. The mixture’s absorbance was measured at 415 nm, and the flavonoid content was expressed as quercetin equivalents (mg QE/100 mg DW).

### Statistical analysis

All the data were statistically analyzed via MSTAT-C software, and the means were separated via Duncan’s multiple range test (*P* < 0.05).

## Results

### Morphological and yield traits

Compared with the control group, the garlic plants treated with the experimental treatments achieved better growth and higher yield. The results demonstrated a substantial influence of *Trichoderma* colonization, chitosan nanoparticle application, and their combination on garlic plants, as illustrated in Table 2. Compared with untreated plants, garlic plants treated with *T. harzianum* and/or chitosan nanoparticles presented improved morphological characteristics (plant height, bulb diameter, clove count, and bulb fresh and dry weights). Compared with the other treatments, the combination of *Trichoderma* inoculation with 100 ppm chitosan nanoparticle spraying resulted in taller plants and larger, heavier bulbs.

**Table 2:** Effect of *Trichoderma* inoculation and chitosan nanoparticle spraying on morphological and yield traits of Garlic plants

Trichoderma inoculation	Chitosan nanoparticle spray (ppm)			Mean
	0	50	100	
	Plant height (cm)			
Non-inoculated	67.37 e	70.40 d	73.73 c	70.50 B
Inoculated	75.33 bc	77.60 ab	79.77 a	77.57 A
Mean	71.35 C	74.00 B	76.75 A	
	Bulb fresh weight (g)			
Non-inoculated	49.37 e	52.70 d	56.37 c	52.81 B
Inoculated	57.73 c	61.10 b	63.50 a	60.78 A
Mean	53.55 C	56.90 B	59.93 A	
	Bulb diameter (mm)			
Non-inoculated	52.7 c	56.0 bc	58.0 ab	55.6 B
Inoculated	59.0 ab	61.3 a	62.3 a	60.9 A
Mean	55.8 B	58.7 AB	60.2 A	
	Clove number/bulb			
Non-inoculated	13.9 b	14.5 ab	15.7 ab	14.7 A
Inoculated	15.5 ab	16.0 a	16.1 a	15.9 A
Mean	14.7 A	15.2 A	16.0 A	
	Bulb dry weight (g)			
Non-inoculated	17.80 c	18.78 c	20.71 b	19.10 A
Inoculated	21.24 b	22.11 ab	23.81 a	22.38 A
Mean	19.52 B	20.45 B	22.26 A	

### Mineral content

The nutritional value of garlic plants was significantly influenced by the experimental treatments. Compared to the control plants, *Trichoderma* inoculation and chitosan nanoparticle spraying resulted in significant increases in garlic nutritional content (N, K, P, and S), as indicated in Table 3. The plants treated with *Trichoderma* inoculation and 100 ppm chitosan nanoparticle spraying had the highest mineral content, while untreated plants had the lowest.

**Table 3:** Effect of *Trichoderma* inoculation and Chitosan nanoparticle spraying on N, P, K, S, TSS, soluble sugar and vitamin C contents in Garlic plants

Trichoderma inoculation	Chitosan nanoparticle spray (ppm)			Mean
	0	50	100	
	N (mg. g⁻¹)			
Non-inoculated	20.74 d	22.22 cd	23.95 bc	22.30 B
Inoculated	24.63 b	25.42 ab	26.80 a	25.62 A
Mean	22.69 B	23.82 B	25.38 A	
	P (mg. g⁻¹)			
Non-inoculated	3.393 c	3.560 bc	3.823 abc	3.592 B
Inoculated	3.977 abc	4.123 ab	4.230 a	4.110 A
Mean	3.685 A	3.842 A	3.027 A	
	K (mg. g⁻¹)			
Non-inoculated	16.64 c	17.67 bc	18.89 ab	17.73 A
Inoculated	19.13 ab	19.37 ab	20.23 a	19.58 A
Mean	17.89 B	18.52 AB	19.56 A	
	S (mg. g⁻¹)			
Non-inoculated	4.210 b	4.403 b	4.737 ab	4.450 A
Inoculated	4.950 ab	5.137 ab	5.440 a	5.176 A
Mean	4.580 A	4.770 A	5.088 A	

### Photosynthetic pigments

**Fig. 1:**
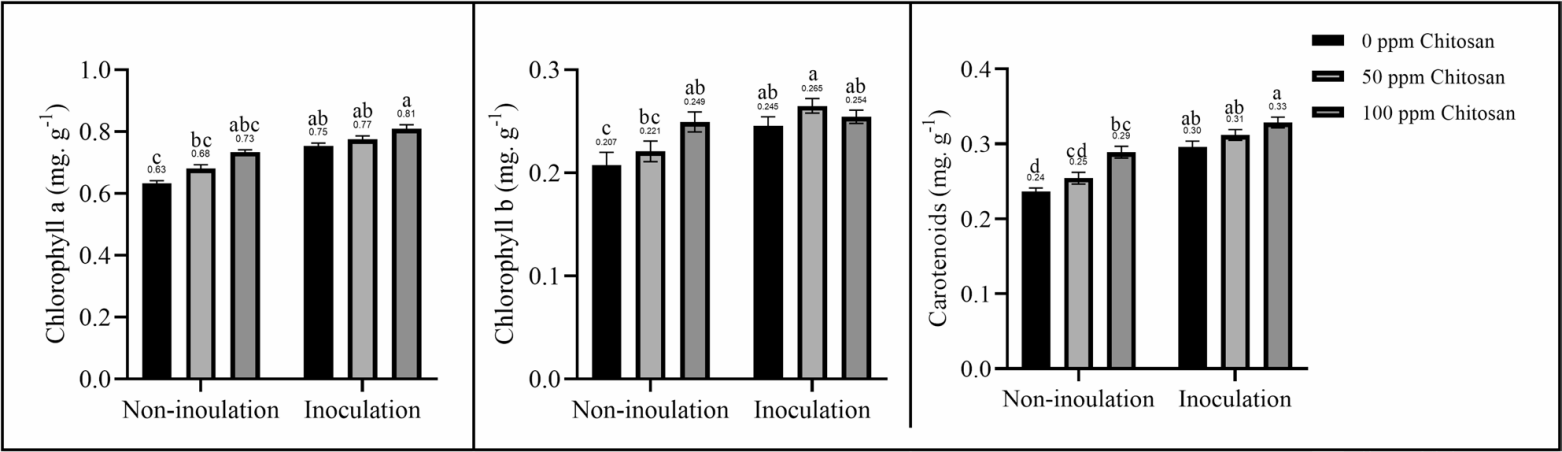
Effect of *Trichoderma* inoculation and chitosan nanoparticle spraying on photosynthetic pigments in garlic plants. Different letters above bars indicate significant differences between treatments using Duncan’s test (*P* < 0.05)

Increasing the photosynthetic pigment content via *T. harzianum* inoculation and the application of chitosan nanoparticles improved the photosynthetic efficiency of garlic plants. Figure 1 shows that the *Trichoderma* and chitosan nanoparticle treatments resulted in considerable increases in the chlorophyll (a, b) and carotenoid levels. The synergistic use of *Trichoderma* inoculation and 100 ppm chitosan nanoparticles significantly increased the content of photosynthetic pigments, resulting in the highest values.

### Soluble sugars and TSS content

**Fig. 2:**
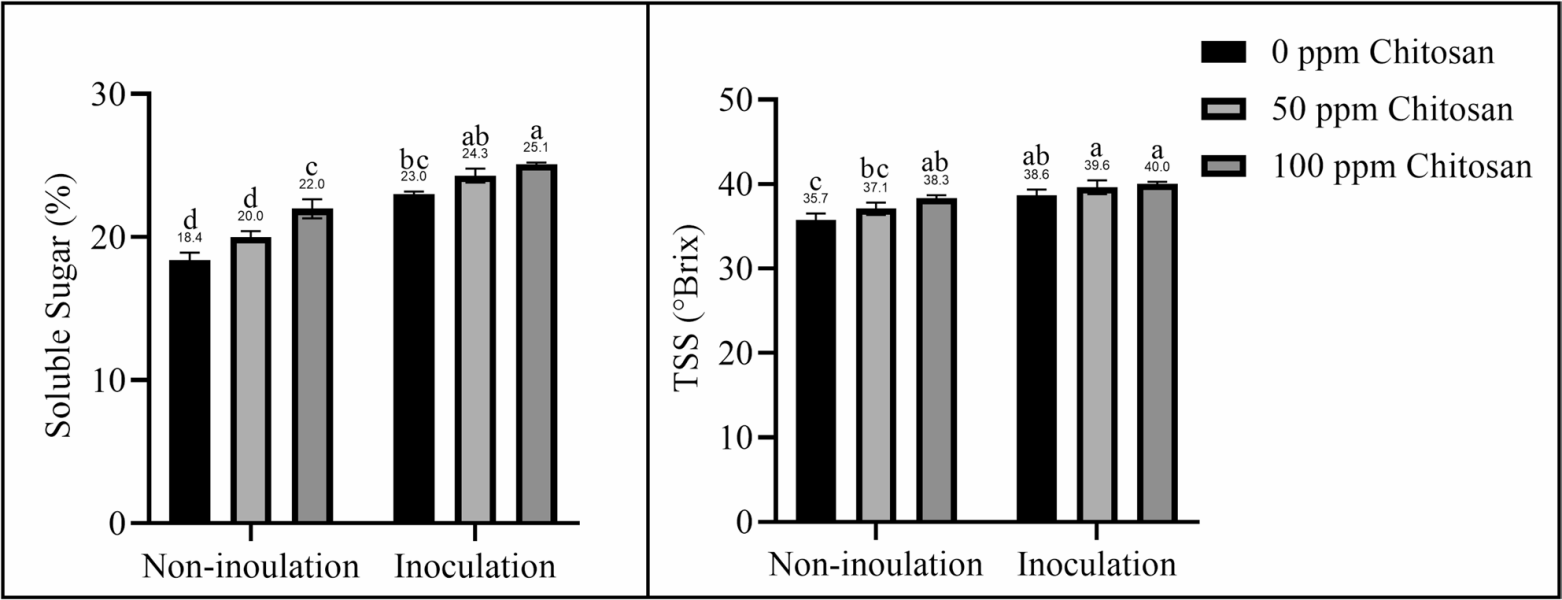
Effect of *Trichoderma *inoculation and chitosan nanoparticle spraying on soluble sugar and TSS in garlic plants. Different letters above bars indicate significant differences between treatments using Duncan’s test (*P* < 0.05)

The experimental treatments significantly affected the accumulation of soluble sugars and TSS in garlic plants. Figure 2 shows that the soluble sugar and TSS contents in garlic cloves tended to increase in response to *Trichoderma* and/or chitosan nanoparticle treatments compared with those in the control plants. The interaction of *Trichoderma* with chitosan nanoparticles significantly increased these parameters. The highest values were found in the combined treatment of plants inoculated with *Trichoderma* and sprayed with 100 ppm chitosan nanoparticles, whereas the lowest values were found in the control plants.

### Antioxidant content

**Fig. 3:**
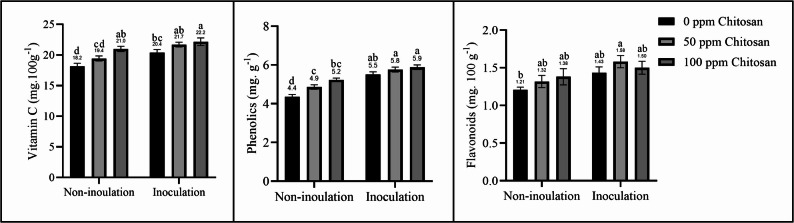
Effect of *Trichoderma* inoculation and chitosan nanoparticle spraying on antioxidant content of garlic plants. Different letters above bars indicate significant differences between treatments using Duncan’s test (*P* < 0.05)

Garlic plants treated with *Trichoderma* and/or chitosan nanoparticles presented increased antioxidant activity and content. Compared with those in the control group, the treated plants presented significantly greater levels of vitamin C, phenols, and flavonoids (Fig. 3). Compared with those in the other treatments, the antioxidant content in the Trichoderma-inoculated plants sprayed with 100 ppm chitosan nanoparticles was greater.

## Discussion

With the growing demand for food and the impact of climate change on environmental resources, it is crucial to adopt agricultural practices that enhance food production while taking into account environmental sustainability. This study demonstrated the positive effects of using natural stimulants, such as chitosan nanoparticles and *Trichoderma* fungi, on plant growth and productivity. Plants inoculated with *T. harzianum* and sprayed with chitosan nanoparticles showed increased nutrient content (Table 3), photosynthetic pigments (Fig. 1), soluble sugars (Fig. 2), and antioxidants (Fig. 3), resulting in improved growth parameters and increased garlic yield (Table 2). However, Sani et al. [1], Liu et al. [7], and Ortega-García et al. [17] reported that *T. harzianum* inoculation can increase soil nutrient availability while reducing the need for chemical fertilizers without significantly affecting plant productivity. In the same context, Sathiyabama and Manikandan [26] and Divya et al. [27] concluded that nanomaterials can reduce the use of agrochemicals and maintain plant productivity. Therefore, this study combined *Trichoderma* inoculation with chitosan nanoparticle spraying, demonstrating this approach to be a useful approach. Several studies have shown the positive effect of *Trichoderma* fungi on the growth and yield of various plants, such as garlic [18], onion [2, 17], potato [19], tomato [1, 9], *Cucurbita pepo* [10], lettuce [13], and *Bupleurum chinense* plants [7]. Chitosan nanoparticle treatment also improved the growth of *Phaseolus vulgaris* [6], tomato [25, 30], *mung bean* [32], *Lupinus termis* [29], wheat [24], rice [27], and *Salvia abrotanoides* [31].

*Trichoderma* fungi improve plant growth performance, physiological functions, and yield via multiple mechanisms. *Trichoderma* can stimulate plant growth and development via an auxin-dependent mechanism, as well as auxin analogues, polyphenol content, and antioxidant activity, thereby improving plant growth, development, and yield [8]. Several studies have also attributed the stimulating effect of *Trichoderma* spp. to the production of growth-promoting substances, such as indole acetic acid [12, 17], auxins, gibberellins [9, 18], siderophores [12], cytokinins, indole butyric acid, salicylic acid, and jasmonic acid [10]. Additionally, *T. harzianum* can improve photosynthetic performance by enhancing the expression of genes involved in photosynthesis-related processes, including the Calvin cycle, increasing the maximal photochemical efficiency of PSII (Fv/Fm), increasing the net photosynthetic rate, and promoting the accumulation of photosynthetic products (total soluble sugar, sucrose, and starch) in leaves and roots [14]. It also modifies the anatomical features of plant leaves, increasing epidermal cell density and stomatal density [20]. Thus, it increases photosynthetic efficiency and accelerates the growth and development of inoculated plants. Soliman et al. [10] and Jamil et al. [12] also found improved photosynthetic parameters (photosynthetic pigment content, gas exchange parameters, chlorophyll fluorescence, and net photosynthetic rate) in plants inoculated with *Trichoderma* fungi.

Soluble sugars are essential osmolytes involved in osmotic adjustment to maintain protoplasm hydration and stabilize cell structure [3]. However, *Trichoderma harzianum* inoculation maintains cell membrane permeability and osmotic potential by increasing the accumulation of osmolytes such as soluble sugars [20]. The beneficial effects of *Trichoderma* fungi in plants are associated with the production of effector metabolites. Trichoderma secondary metabolites (SMs) are low-molecular-weight compounds, including mycotoxins, pigments, and antibiotics, that act synergistically with other compounds to regulate interactions between organisms, induce systemic resistance, and promote plant growth. SMs also activate signaling pathways associated with biotic and abiotic stress resistance, phytohormone biosynthesis, and growth regulation [8], thereby stimulating plant growth, controlling pathogens, and increasing crop yield under various conditions.

*Trichoderma* inoculation improves soil structure, fertility, and health by lowering soil pH [10]– [11], activating soil enzymes [7, 14], secreting organic acids [1, 14], increasing the abundance, diversity, and activity of beneficial bacterial and fungal communities [7, 21], decomposing organic matter [4], solubilizing soil phosphorus [10, 15, 17, 21], and enhancing the availability and uptake of nutrients such as N, P, K, Mg, Mn, and Fe [7, 10, 17, 22]. *Trichoderma* fungi convert insoluble nutrients into a readily available form by solubilization, exchange reactions, acidification, and chelation [15]. *Trichoderma* produces metal chelators, such as siderophores, and releases them into the rhizosphere to dissolve and chelate minerals, making nutrients available to plants, in addition to their important role in biocontrol, biodegradation, and growth promotion [8]. Furthermore, *Trichoderma harzianum* has been shown to improve root growth and function. Plant growth hormones, such as IAA and GA, produced by *Trichoderma* fungi directly promote root cell proliferation and expansion [14, 15], which in turn enhances root system architecture and significantly improves root growth parameters (fresh and dry weights, length, surface area, volume, and viability) [11, 12, 14, 20, 22]. Thus, it improves root function in terms of water and nutrient uptake and transport [7, 9, 20] and reduces fertilizer requirements of inoculated plants [11, 17, 22]. It also reduces pathogenic microorganisms in the soil [18, 19, 21]. Therefore, *Trichoderma* fungi are recommended as biofertilizers and biopesticides.

Plants in arid areas are exposed to various biotic and abiotic stresses. However, *Trichoderma* fungi have the capacity to induce systemic resistance against adverse conditions. *Trichoderma* exhibits physiological flexibility and environmental adaptability, functioning as an effective stimulant that induces plant defense responses by triggering antioxidant mechanisms. Thus, plants inoculated with *Trichoderma* spp. present increased contents of antioxidants, such as proline, vitamin C, flavonoids, phenols, and enzymes, which reduce the levels of toxic compounds and maintain the integrity, structure, and function of the photosynthetic apparatus and cell membrane [2, 9, 10, 16].

Furthermore, phenolic compounds accumulated in *T. asperellum-*inoculated plants may function as electron and hydrogen donors, protecting root tissues from pathogen-induced oxidative stress [17]. Soil-borne diseases cause significant damage to garlic crops before and after harvest worldwide, reducing the quality and quantity of marketable garlic bulb production [48]. However, *Trichoderma* fungi can confer protection against soil-borne disease through several mechanisms, including competition for nutrients; induction of systemic resistance; antibiosis; and mycoparasitism [16], as well as the secretion of cell wall-degrading enzymes, such as proteases and chitinases, which have antifungal properties and act as elicitors of plant defense systems to suppress plant pathogens and help control plant diseases [12].

Plants in arid environments have evolved physiological adaptations to survive, particularly in response to climate change, such as the accumulation of organic solutes and increased antioxidant activity [31]. However, chitosan nanoparticles can improve the effectiveness of these mechanisms, modifying nutritional, physiological, biochemical, and anatomical features by accumulating organic osmolytes such as amino acids, TSS, carbohydrates, and proline, providing osmotic adjustment and protecting cell membranes [6, 29]. The negative surface charge, fine size, and extensive surface area of chitosan nanoparticles enhance their absorption, transport, and working efficiency [26]. Thus, it increases plant nutrient uptake [6, 25].

Photosynthetic pigments are a key factor in determining the efficiency of photosynthesis. However, plants grown in arid regions suffer from disturbed water relations, low chlorophyll contents, and reduced photosynthesis [49]. However, chitosan nanoparticles can improve photosynthetic capacity and efficiency by increasing photosynthetic pigments, stomatal conductance, and CO₂ fixation, as well as improving water use efficiency [24, 29, 31]. The stimulatory effect of chitosan nanoparticles in plants is associated with elevated levels of phytohormones, such as indoleacetic acid, cytokinin, and gibberellic acid [27, 29]. In addition, it stimulates signal transduction pathways involving nitric oxide and hydrogen peroxide, which activate antioxidant mechanisms [6] and increase the content of antioxidants, including carotenoids, proline, phenols, flavonoids, and antioxidant enzymes [29, 32]. It activates defense responses, enhances water use efficiency, protects cell walls and membranes, maintains the photosynthetic system, enhances plant immune responses, and mitigates adverse conditions. Thus, it improves physiological responses and maintains plant productivity in arid regions [6, 18, 31, 32]. In addition to their antioxidant action, chitosan nanoparticle-treated plants exhibit fortified cell walls and reduced levels of toxic compounds, which increase resistance to pathogens and other stresses and lead to better growth performance; improved morphological, metabolic, and physiological features; and increased crop yields [6, 25, 32].

The combination of chitosan nanoparticles and *Trichoderma* fungi has proven to be extremely beneficial. The chitosan nanoparticles exerted a stimulating effect on the *Trichoderma*, improving soil health and positively impacting plant growth [33, 34]. The combined use of *Trichoderma* fungi and chitosan nanoparticles stimulates *Trichoderma* activity and increases chitinase production, thereby enhancing pathogen control and promoting plant growth and yield [33]. This increases the productivity of garlic plants while reducing the use of agrochemicals. Therefore, *Trichoderma* biofertilizer can be recommended for commercial production [7]. Overall, *Trichoderma* inoculation and chitosan nanoparticle spraying work synergistically to create favorable conditions for plant growth by increasing nutrient availability and organic osmolyte accumulation, improving photosynthetic capacity and efficiency, producing growth-promoting substances, activating the antioxidant system, and maintaining plant health, which modulates physiological and biological processes that result in improved plant growth and increased crop yield.

## Conclusion

This study revealed that *Trichoderma asperellum* inoculation and chitosan nanoparticle spraying can be sustainable and effective agricultural practices that successfully increase garlic growth and yield by increasing nutrient uptake and photosynthetic pigments, increasing the accumulation of organic solutes, and activating the antioxidant system. The combination of *Trichoderma* inoculation and 100 ppm chitosan nanoparticle spraying achieved the best results, improving growth performance, nutritional qualities, and yield traits. Further experiments are needed to develop and optimize nanoparticle formulations for foliar compatibility, as well as to ascertain the effect of nanoparticles on soil microorganisms.

## Supplementary Information

Supplementary Material 1

## Data Availability

All data are included in the manuscript.
