# Supplementary material for: Synergistic effect of Trichoderma harzianum and chitosan nanoparticles on garlic plants in arid regions
Source: BMC Plant Biol. 2025 Dec 12;26:12. doi: 10.1186/s12870-025-07844-5 (PMC12766955; doi:10.1186/s12870-025-07844-5)
Supplement: Supplementary file 1 — Supplementary Material 1 [file 12870_2025_7844_MOESM1_ESM.pptx]

## Slide 1
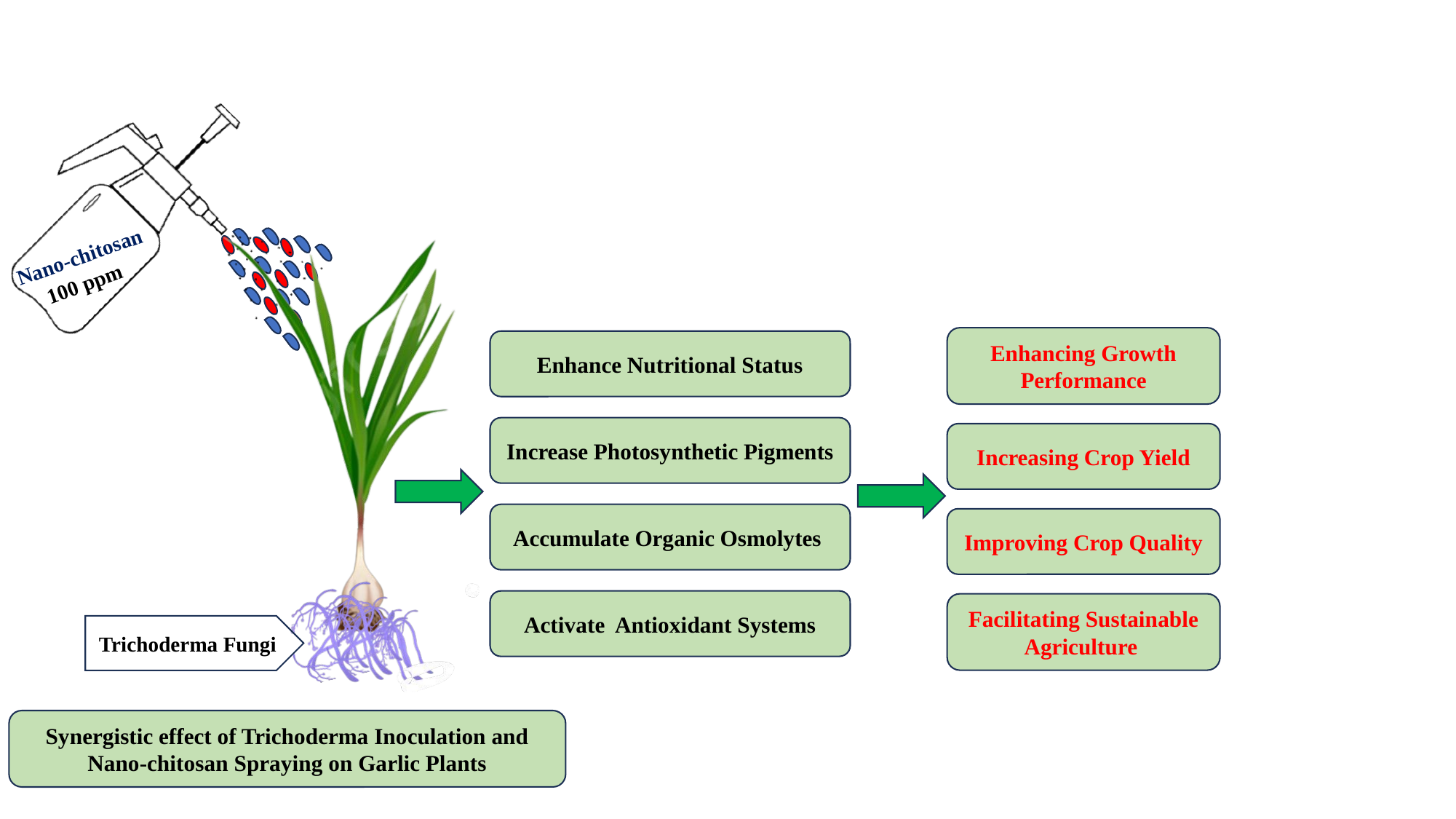

Nano-chitosan
100 ppm
Enhancing Growth Performance
Enhance Nutritional Status
Increase Photosynthetic Pigments
Increasing Crop Yield
Accumulate Organic Osmolytes
Improving Crop Quality
Activate Antioxidant Systems
Facilitating Sustainable Agriculture
Trichoderma Fungi
Synergistic effect of Trichoderma Inoculation and Nano-chitosan Spraying on Garlic Plants
